# Comparison of Histological Skin Changes After Massive Weight Loss in Post-bariatric and Non-bariatric Patients

**DOI:** 10.1007/s11695-024-07066-y

**Published:** 2024-01-26

**Authors:** Mohamed Hany, Ahmed Zidan, Nasser A. Ghozlan, Mohamed N. Ghozlan, Anwar Ashraf Abouelnasr, Eman Sheta, Yasser Hamed, Hassan Kholosy, Mohammed Soffar, Walid M. El Midany, Bart Torensma

**Affiliations:** 1 Department of Surgery, Medical Research Institute, Alexandria University, 165 Horreya Avenue, Hadara, Alexandria, 21561 Egypt; 2 Madina Women’s Hospital, Alexandria University, Alexandria, Egypt; 3 Plastic and Reconstructive Surgery - Alexandria University, Alexandria, Egypt; 4 Department of Plastic Surgery, Alexandria University, Alexandria, Egypt; 5 Pathology Department, Faculty of Medicine, Alexandria University, Alexandria, Egypt; 6 Medical Insurance Organization, Alexandria, Egypt; 7 Leiden University Medical Center (LUMC), Leiden, The Netherlands

**Keywords:** Skin changes, Massive weight loss, Bariatric metabolic surgery, Non-surgical weight loss, Body-contouring surgery, Skin histology, Epidermal thickness, Collagen, Elastin

## Abstract

**Background:**

Changes in the skin structure, including the collagen and elastin content, have been reported with massive weight loss (MWL) following bariatric metabolic surgery (BMS) and have been correlated to a higher risk of complications after body-contouring surgery (BCS). This study aimed at comparing the histological characteristics of the skin of patients having surgical MWL (SMWL) post-BMS to those with non-surgical massive weight loss (NSMWL).

**Methods:**

This prospective study compared the epidermal thickness, and collagen and elastin fibers content in 80 skin biopsies obtained from BCS procedures performed to patients who experienced MWL defined more than 50% of excess weight loss (%EWL) either SMWL (40 biopsies) or NSMWL (40 biopsies). Twenty biopsies in each group were obtained from abdominoplasties and 20 from breast reductions. Epidermal thickness was measured in H&E-stained sections, collagen fibers were assessed using Masson trichrome-stained sections, and elastin fibers were assessed using Modified Verhoeff’s stained sections. Image analysis software was used to calculate the fractions of collagen and elastin fibers.

**Results:**

This study included 77 patients, 38 SMWL patients, and 39 NSMWL patients. The SMWL group had a significantly higher age (*p* < 0.001), a longer time interval from intervention (*p* < 0.001), higher initial weight (*p* < 0.001), higher initial BMI (*p* < 0.001), lower current weight (*p* = 0.005), lower current BMI (*p* < 0.001), and significantly higher %EWL than NSMWL group (*p* < 0.001). No significant differences were detected between the two groups regarding complications after abdominoplasty (*p* = 1.000). The elastic fibers content in the dermis was significantly higher in the abdominal region of the NSMWL group than SMWL (*p* = 0.029). All other parameters showed non-significant differences between NSMWL and SMWL in the skin of abdomen and breast.

**Conclusion:**

The SMWL group had a significant reduction in elastic fiber content in the skin of the abdomen compared to the NSMWL group. The collagen content was equally reduced in both groups with non-significant differences in both breast and abdomen regions in both groups.

**Graphical Abstract:**

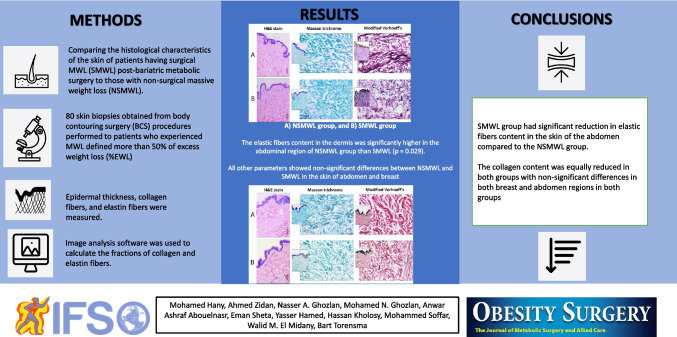

## Introduction

Bariatric metabolic surgery (BMS) is currently the most effective treatment for severe obesity, offering significantly greater massive weight loss (MWL) compared to non-surgical interventions, characterized by its rapidity, progression, and sustainability [1]. However, this rapid and substantial weight reduction frequently leads to the possible development of excessive loose skin. This condition poses both psychological and physical challenges for patients, often necessitating subsequent body-contouring surgery (BCS) [2–4]. High incidences of BCS have been observed in patients following BMS, with reported rates varying from 6 to 21%. Among these procedures, abdominoplasty is the most performed, followed by mastopexy [4–7]. Furthermore, patients who undergo BCS following BMS are reported to have a higher risk of developing complications compared to other patients [8–10]. BMS leads to significant changes in skin structure, notably impacting the collagen and elastin fibers within the dermis layer. These fibers are essential for maintaining skin integrity, strength, and elasticity. Rapid and substantial MWL post-BMS often results in changes in these critical skin components. These changes compromise the skin’s ability to retract and conform to the body’s new contours, thereby elevating the risk of suboptimal early and late outcomes in BCS [3, 7]. Nevertheless, few studies have specifically addressed the changes in skin structure associated with significant MWL. To the best of our knowledge, no research to date has directly compared the collagen and elastin content in the skin dermis of patients who have undergone surgical MWL (SMWL), following BMS with those who have undergone non-surgical MWL (NSMWL) following non-surgical interventions such as diet and exercise. Consequently, this study was designed to prospectively compare the collagen and elastin content in addition to the epidermal thickness of the skin of patients who have experienced SMWL with those who have NSMWL.

## Methods

This prospective cohort study enrolled patients undergoing BCS following SMWL or NSMWL between March 2021 and October 2023 in Madina Women’s Hospital and Main University Hospital, Alexandria, Egypt. The study was conducted in accordance with the principles of the Declaration of Helsinki and approved by the ethical committee board. All patients signed informed consent. The study was approved by the ethical committee under registration number 0305862. Forty skin samples, 20 from breast skin and 20 from abdominal skin, were prospectively collected from the SMWL patients upon abdominoplasty and breast reduction procedures, and similarly 40 samples from NSMWL patients.

### Study Endpoints

The primary endpoint of this study is to compare the epidermal thickness, collagen, and elastin content in the skin of individuals who have undergone SMWL to those NSMWL.

### Inclusion Criteria

Patients, aged between 18 and 60 years, and an initial body mass index (BMI) of ≥ 35 kg/m^2^ before weight loss intervention who achieved MWL defined as a percentage of excess weight loss (%EWL) of ≥ 50% [11].

### Exclusion Criteria

Factors associated with skin aging such as history of smoking, diabetes mellitus, metabolic syndrome, prolonged exposure to sunlight or ultraviolet rays, rheumatological diseases, and prolonged use of steroids [11–13].

### Data Collection

Demographic data such as age, gender, time interval from the weight loss intervention to BCS, and associated medical problems before and after MWL. Weight loss data which included method of weight loss either SMWL or NSMWL, initial weight and BMI, weight, and BMI before BCS, and %EWL were collected. Complications of BCS were recorded with the Clavien-Dindo classification [14]. Furthermore, histological examination of the skin biopsies included epidermal thickness, and percentage of collagen and elastic fibers in biopsies.

### Skin Examination

Skin biopsies were obtained from the removed skin during abdominoplasty and breast reduction procedures involving the epidermis, dermis, and subcutaneous tissue. Skin biopsies from both groups were fixed in 10% buffered formalin for 24 h. The following day, sections were taken and placed in plastic cassettes. The biopsy was well oriented in the cassette to show the epidermis overlying the dermis and subcutaneous tissue. Cassettes were processed in different grades of alcohol followed by xylene until embedded in paraffin. Three serial five-micron-thick sections were cut and mounted on glass slides. The first was stained by H&E staining using standard protocols. The second slide was stained by Masson’s trichrome stain (Trichrome Staining Kit (Modified Masson’s), ScyTeh, USA). Meanwhile, the last slide was stained by elastic stain (Elastic Stain Kit (modified Verhoeff’s), USA).

**Fig. 1:**
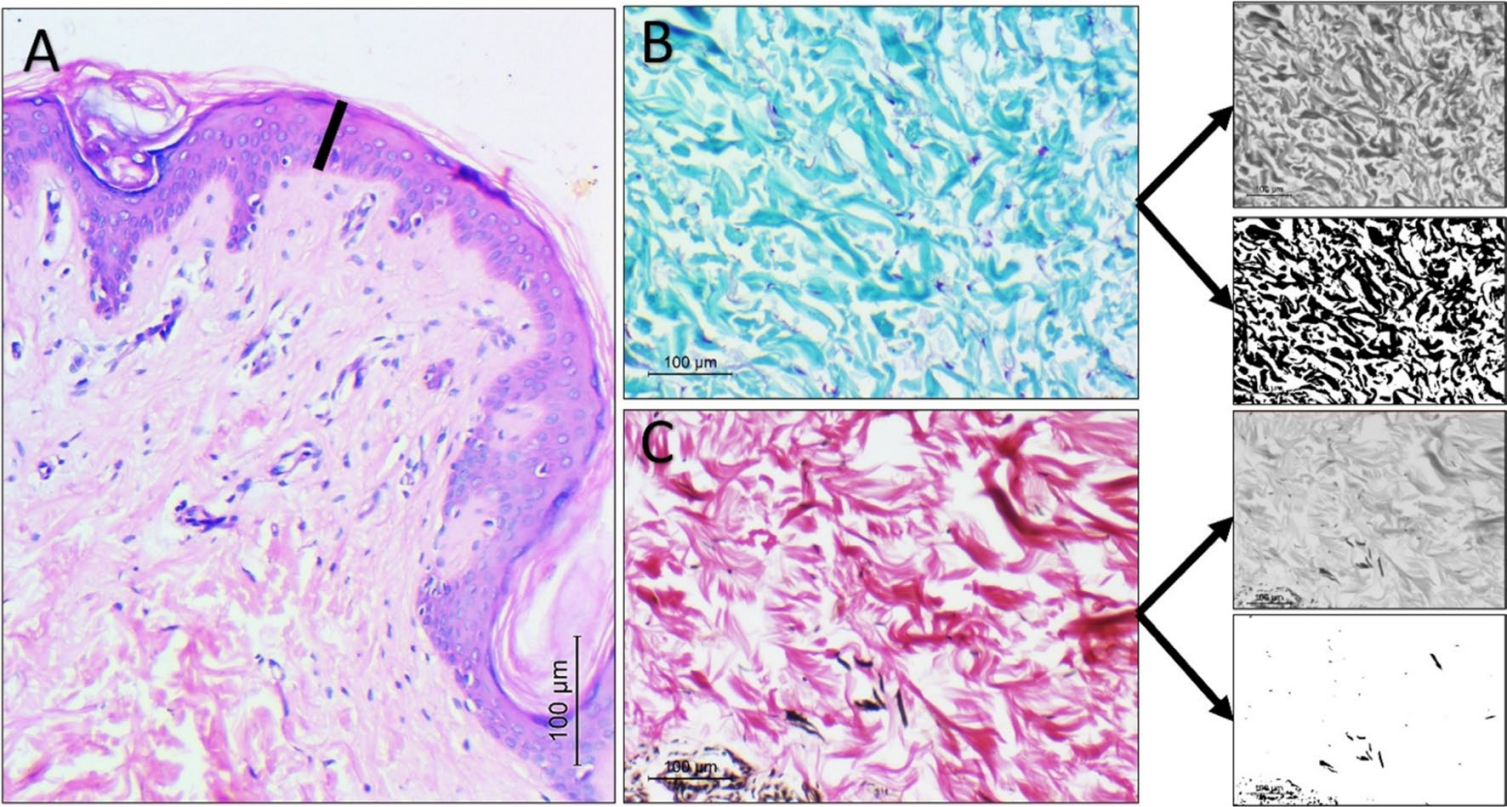
Assessment of different histopathologic skin parameters in this study at × 200 power (scale bar = 100 microns): **A** H&E-stained section to measure the epidermal thickness (black line). **B** Masson trichrome-stained section to assess collagen bundles which stained blue. The photo is changed into gray scale image (upper photo); then, segmentation of color was done to assess percentage of collagen fibers (lower photo) **C** Modified Verhoeff’s stained section to assess the elastic fibers. Collagen bundles are stained deep pink while elastic fibers are seen black in color. The photo was changed into gray scale 8-bit image (upper photo); then, segmentation of color was done to measure the percentage of elastic fibers (lower photo)

**Fig. 2:**
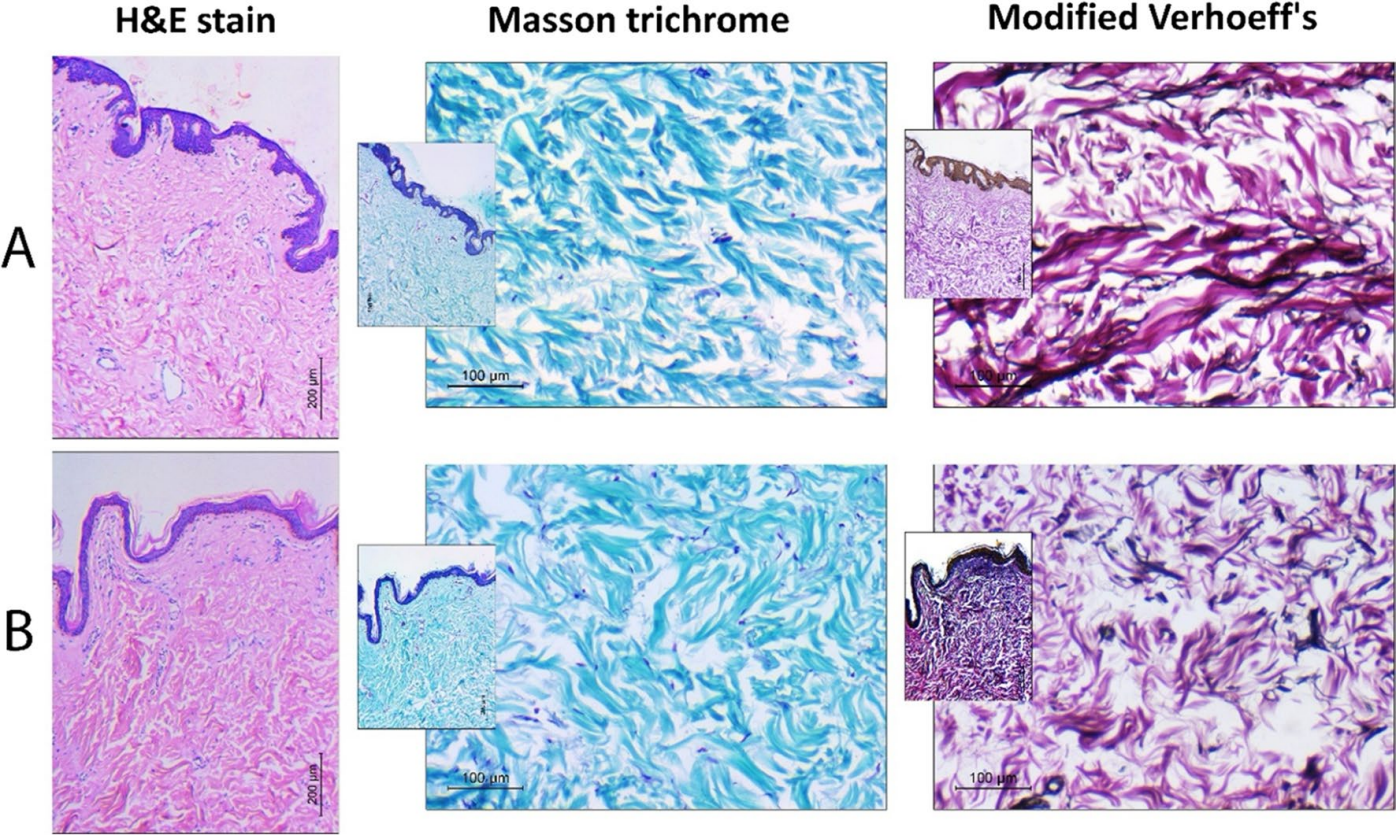
Histopathologic specimens of abdominal skin biopsies in studied cases: **A** NSMWL group and **B** SMWL group. H&E-stained sections (× 100, scale bar = 200 microns) shows no difference in epidermal thickness in both groups. Masson trichrome staining (× 200, inset: × 100) shows blue stained collagen bundles in the dermis. Modified Verhoeff’s stain (× 200, inset: × 100) shows deep pink collagen bundles and black-colored elastic tissues. NSMWL group shows increased elasticity in comparison to the SMWL group

**Fig. 3:**
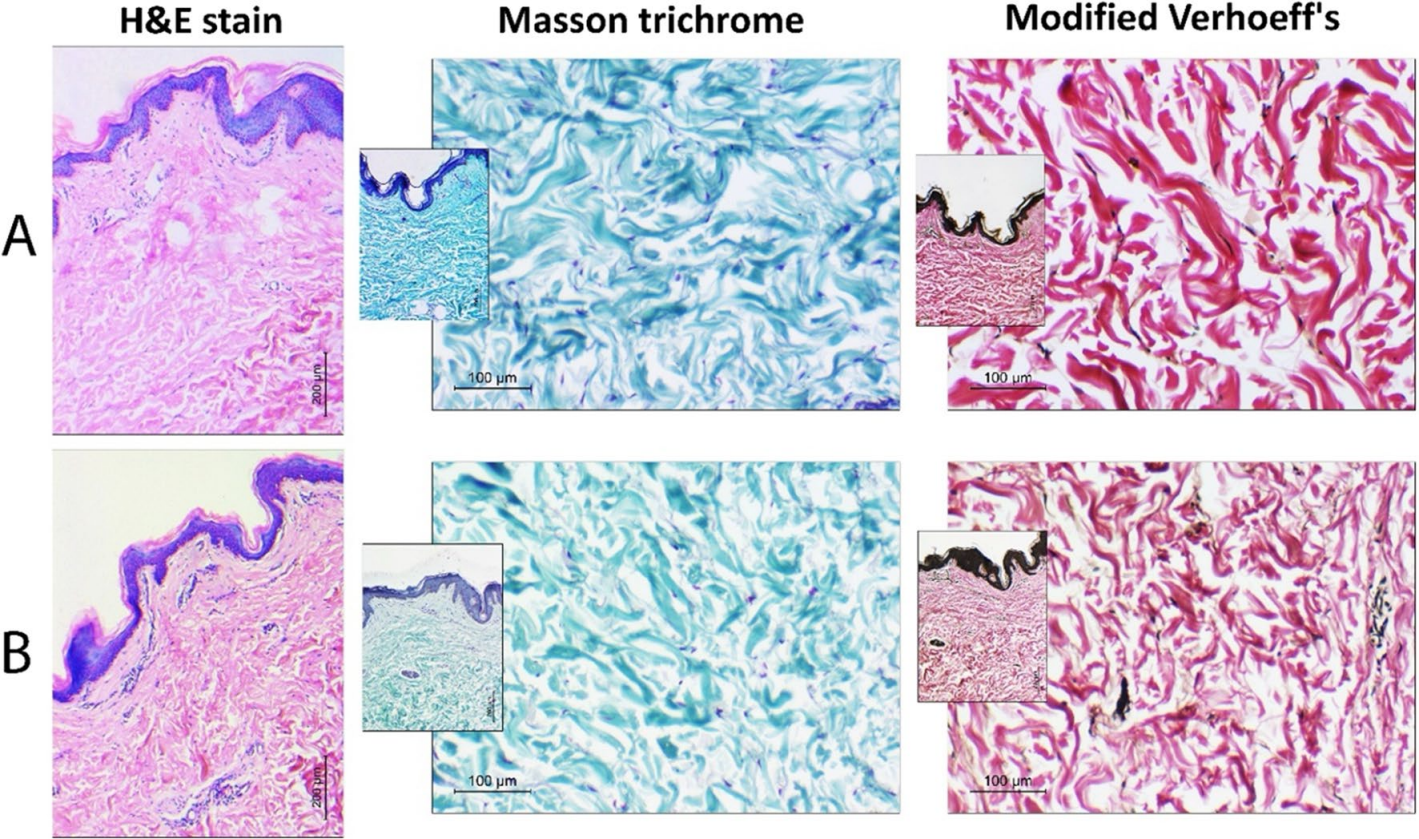
Histopathologic assessment of breast skin biopsies in studied cases: **A** NSMWL group and **B** SMWL group. H&E-stained sections (× 100, scale bar = 200 microns) shows no difference in epidermal thickness in both groups. Masson trichrome staining (× 200, inset: × 100) shows blue-stained collagen bundles in the dermis. Modified Verhoeff’s stain (× 200, inset: × 100) shows deep pink collagen bundles and black-colored elastic tissues. No statistically difference in collagen and elastic tissues were detected between both groups

H&E-stained sections were examined by light microscope to exclude the presence of any pathology; then, multiple photos of the epidermis were taken using a camera coupled to a microscope. Photos were taken at × 200 power, and using the image analysis software, Image J (http://imagej.net), epidermal thickness was measured from the surface of the stratum corneum to the basement membrane. At least four measurements were taken on each photo; then, the mean was calculated. On Masson’s trichrome-stained sections, collagen fibers were stained blue. Multiple photos at × 200 power were taken from the dermis. Meanwhile, in modified Verhoeff’s stained sections, collagen bundles were seen deep pink in color while elastic tissue was seen black. Areas rich in elastic tissue were photographed at × 200 power. The elastic lamina of dermal vessels was stained black and considered as an internal positive control [15]. Photos in both Masson’s trichrome and modified Verhoeff’s stained slides were taken from the dermis and were assessed using Image J software. Photos were changed into gray scale 8-bit images; then, the threshold was adjusted to highlight the desired shade. The area of collagen or elastic tissue was calculated as a percentage/fraction of the total examined areas [16]. All biopsies were assessed blindly without prior knowledge of the patient’s history or clinical data. Areas of technical artifacts or large pilosebaceous structures were avoided (Figs. 1, 2, 3).

### Statistical Analysis

Descriptive and inferential statistics were used for the analyses. All data were tested for normality using the Kolmogorov–Smirnov, Q-Q plot, and Levene’s tests. Categorical variables are expressed as numbers and percentages. Normally and non-normally distributed continuous variables are presented as means with standard deviations (SDs) and medians with interquartile ranges. When appropriate, categorical variables were tested using Pearson’s chi-square or Fisher’s exact test. Normally distributed continuous data were tested with independent samples with Student’s *t*-test or the Mann–Whitney *U* test was used for skewed (nonparametric) data. A significant level of 0.05 was used in all analyses. All analyses were conducted using R-software (version 4.2.2. Vienna, Austria).

Multiple linear regression analyses were conducted to estimate the effect of age, gender, type of weight loss intervention, type of body contour surgery, %EWL, time elapsed since intervention, post-BCS complications on elastic fibers proportions, collagen fibers proportion, and epidermal thickness.

### Sample Size

The sample size calculation was done using the “pwr” package version 1.3–0. A large effect size (Cohen’s *D*) of 0.9 was used for the difference in collagen%, elastic%, and epidermal thickness between the control and bariatric using *t* test and a power of 80% with a significance level of 0.05; this resulted in a minimum sample size of 20 patients in each subgroup.

## Results

This study included a total of 77 patients, with 38 post-bariatric patients in the SMWL group, and 39 patients in the NSMWL group. Three patients (3.9%) had concomitant abdominoplasty and breast reduction and were excluded.

### Demographic and Personal Characteristic Data

The SMWL group had a significantly higher age mean ± sd (41.4 ± 6.2 vs. 30.3 ± 4.3, *p* < 0.001), a longer time interval from intervention to the BCS (23.7 ± 5.6 vs. 18.7 ± 2.8 months, *p* < 0.001), higher initial weight (112.7 ± 5.7 vs. 98.6 ± 7.4, *p* < 0.001), higher initial BMI (40.3 ± 1.9 vs. 35.7 ± 1.3, *p* < 0.001), lower current weight (78.1 ± 5.9 vs. 82.2 ± 6.6, *p* = 0.005), and lower current BMI (27.9 ± 0.8 vs. 29.8 ± 0.9, *p* < 0.001). Moreover, the SMWL group had significantly higher %EWL compared to the NSMWL group (80.9% ± 5.6 vs. 55.5% ± 6.7, p < 0.001) (Table 1).

**Table 1:** Distribution of demographic and personal characteristics of the study participants

	NSMWL (*n* = 39)	SMWL (*n* = 38)	*p*
Age, mean ± SD	30.3 ± 4.3	41.4 ± 6.2	< 0.001*
Gender			
Female, *n* (%)	34 (87.2%)	32 (84.2%)	0.963
Male, *n* (%)	5 (12.8%)	6 (15.8%)	
Time interval from intervention (months), mean ± SD	18.7 ± 2.8	23.7 ± 5.6	< 0.001*
Initial weight, mean ± SD	98.6 ± 7.4	112.7 ± 5.7	< 0.001*
Initial BMI, mean ± SD	35.7 ± 1.3	40.3 ± 1.9	< 0.001*
Current weight, mean ± SD	82.2 ± 6.6	78.1 ± 5.9	0.005*
Current BMI, mean ± SD	29.8 ± 0.9	27.9 ± 0.8	< 0.001*
%EWL, mean ± SD	55.5 ± 6.7	80.9 ± 5.6	< 0.001*
Associated medical problems before weight loss			
HTN, *n* (%)	4 (10.3%)	5 (13.2%)	0.737
Asthma, *n* (%)	2 (5.1%)	1 (2.6%)	1.000
Sleep apnea, *n* (%)	2 (5.1%)	2 (5.3%)	1.000
Osteoarthritis, *n* (%)	1 (2.6%)	3 (7.9%)	0.358
Dyslipidemia, *n* (%)	2 (5.1%)	2 (5.3%)	1.000
Cardiac ischemia, *n* (%)	0 (0.0%)	1 (2.6%)	0.494
Polycystic ovary, *n* (%)	2 (5.1%)	1 (2.6%)	1.000
Associated medical problems after weight loss			
HTN, *n* (%)	2 (5.1%)	2 (5.3%)	1.000
Asthma, *n* (%)	1 (2.6%)	0 (0.0%)	1.000
Sleep apnea, *n* (%)	0 (0.0%)	0 (0.0%)	1.000
Osteoarthritis, *n* (%)	0 (0.0%)	0 (0.0%)	1.000
Dyslipidemia, *n* (%)	1 (2.6%)	1 (2.6%)	1.000
Cardiac ischemia, *n* (%)	0 (0.0%)	0 (0.0%)	1.000
Polycystic ovary, *n* (%)	0 (0.0%)	0 (0.0%)	1.000

*NSMWL*, non-surgical massive weight loss; *SMWL*, surgical massive weight loss

^*^Statistically significant (*p* < 0.05)

### Weight Loss Interventions

The BMS in the SMWL group included 27 (71.1%) laparoscopic sleeve gastrectomy procedures, six one-anastomosis gastric bypass (15.8%), and five Roux-en-Y gastric bypass (31.2%) procedures. The non-surgical interventions included hypo-energetic diet with exercise in 25 patients (64.1%), hypo-energetic diet alone in eight (20.5%), and hypo-energetic diet with orlistat medications in six (15.4%).

### BCS Procedures

There were no significant differences between the two groups (NSMWL vs SMWL) regarding complications after abdominoplasty (CDI 15 vs. 10%, CDIIIa 5 vs. 10%), nor breast reduction procedures (CDI 5 vs. 10%, CDIIIa 10 vs. 5%) (*p* = 1.000) (Table 2).

**Table 2:** Complications after abdominoplasty and breast reduction procedures

	Abdominoplasty (*n* = 40)		*p*	Breast reduction (*n* = 40)		*p*
	NSMWL (*n* = 20)	SMWL (*n* = 20)		NSMWL (*n* = 20)	SMWL (*n* = 20)	
Complications after BCS						
Seroma, *n* (%)	2 (10%)	1 (5%)	1.000	1 (5%)	1 (5%)	1.000
Hematoma, *n* (%)	1 (5%)	1 (5%)	1.000	0 (0%)	1 (5%)	1.000
Wound infection, *n* (%)	0 (0%)	0 (0%)	1.000	1 (5%)	1 (5%)	1.000
Wound infection requiring debridement), *n* (%)	0 (0%)	0 (0%)	1.000	0 (0%)	1 (5%)	1.000
Seroma requiring drainage, *n* (%)	0 (0%)	1 (5%)	1.000	1 (5%)	1 (5%)	1.000
Wound dehiscence, *n* (%)	1 (5%)	1 (5%)	1.000	0 (0%)	0 (0%)	1.000
Clavien-Dindo classification of complications after BCS						
I, *n* (%)	3 (15%)	2 (10%)	1.000	1 (5%)	2 (10%)	1.000
II, *n* (%)	0 (0%)	0 (0%)	1.000	0 (0%)	1 (5%)	1.000
IIIa, *n* (%)	1 (5%)	2 (10%)	1.000	2 (10%)	1 (5%)	1.000

*NSMWL*, non-surgical massive weight loss; *SMWL*, surgical massive weight loss

### Histological Examination of Skin Biopsies

The thickness of the skin epidermis in micrometers and fractions of collagen and elastic fibers in skin biopsies from the NSMWL and SMWL groups were measured. Only elastic fiber content in the dermis was significantly higher in the abdominal region of the NSMWL group than SMWL (*p* = 0.029). All other parameters showed non-significant differences between NSMWL and SMWL in the skin of the abdomen and breast (Table 3).

**Table 3:** Histological characteristics of skin biopsies from abdomen and breast in NSMWL and SMWL groups

	Abdominoplasty biopsies (*n* = 40)		*p*	Breast reduction biopsies (*n* = 40)		*p*
	NSMWL (*n* = 20)	SMWL (*n* = 20)		NSMWL (*n* = 20)	SMWL (*n* = 20)	
Skin epidermal thickness (in μm), mean ± SD	60.3 ± 17.7	56.7 ± 12.9	0.472	56.1 ± 15.1	53.1 ± 7.0	0.438
Collagen fibers content (%), mean ± SD	49.5% ± 4.2	49.2% ± 4.6	0.801	49.6% ± 5.4	49.3% ± 5.2	0.855
Elastic fibers content (%), mean ± SD	6.8% ± 2.4	5.0% ± 2.4	0.029*	6.7% ± 2.4	6.3% ± 2.3	0.562

*NSMWL*, non-surgical massive weight loss; *SMWL*, surgical massive weight loss

Collagen and elastic fiber contents were calculated as percentage of total examined areas

^*^Statistically significant (*p* < 0.05)

Table 4 and Table 5 present the results of multiple linear regression analyses, exploring the impact of various factors on key skin structure-related outcomes, including elastic fibers proportion, collagen fibers proportion, and epidermal thickness. The examined factors include age, gender, type of weight loss intervention, type of BCS, %EWL, time elapsed since intervention, and post-BCS complications. Significant predictors of elastic fibers proportion were the type of weight loss intervention and time elapsed from intervention. SMWL was linked to a decrease in elastic fibers proportion compared to NSMWL (estimate =  − 3.56, 95% CI − 6.41 to − 0.70, *p* = 0.016), whereas the time elapsed since intervention exhibited a significant positive association with elastic fibers proportion, indicating that as time passes post-intervention, elastic fibers proportion tends to increase (estimate = 0.17, 95% CI 0.03 to 0.30, *p* = 0.016) (Table 4). On the contrary, SMWL was associated with higher collagen fiber proportion compared to NSMWL (estimate = 5.94, 95% CI 2.78 to 11.49, *p* = 0.036) (Table 4). In addition, higher collagen fiber proportion was associated with higher seroma drainage after BCS compared to no complications post-BCS (estimate = 9.96, 95% CI 4.65 to 19.25, *p* = 0.036) (Table 4). Hematoma formation (30.32, 95% CI 13.72 to 46.93, *p* = 0.001) and drainage of seroma (30.50, 95% CI 4.52 to 56.47, *p* = 0.022) were significantly associated with increased epidermal thickness (Table 4 and 5).

**Table 4:** Multiple linear regression analyses for the effect of age, gender, type of weight loss intervention, type of BCS, %EWL, time elapsed since intervention, and post-BCS complications on elastic and collagen fibers proportions

Predictors	Estimate	SE	95% CI		*p*
			Lower	Upper	
Predictors of elastic fibers proportion					
(Intercept)	0.16	3.37	− 6.57	6.89	0.962
Age	0.02	0.06	− 0.09	0.13	0.707
Gender					
Female (reference)					
Male	0.16	0.95	− 1.74	2.06	0.865
Weight loss intervention					
Non-surgical (reference)					
Surgical	− 3.56	1.43	− 6.41	− 0.70	0.016*
Type of body contour surgery					
Abdominoplasty (reference)					
Breast reduction	0.42	0.57	− 0.72	1.56	0.465
%EWL	0.05	0.04	− 0.04	0.14	0.241
Time elapsed since intervention	0.17	0.07	0.03	0.30	0.016*
Post-BCS complications					
No complications (reference)					
Dehiscence debridement and sutures	− 0.51	1.72	− 3.94	2.93	0.769
Hematoma	− 1.86	1.53	− 4.92	1.20	0.230
Seroma	− 1.45	1.09	− 3.63	0.72	0.187
Seroma drainage	− 2.51	2.40	− 7.29	2.28	0.300
Wound infection antibiotics	3.41	2.43	− 1.43	8.26	0.164
Wound infection debridement	− 0.50	1.43	− 3.36	2.36	0.729
Predictors of collagen fibers proportion					
(Intercept)	62.89	6.54	49.83	75.95	< 0.001*
Age	− 0.16	0.11	− 0.38	0.05	0.134
Gender					
Female (reference)					
Male	0.02	1.84	− 3.66	3.70	0.992
Weight loss intervention					
Non-surgical (reference)					
Surgical	5.94	2.78	0.39	11.49	0.036*
Type of body contour surgery					
Abdominoplasty (reference)					
Breast reduction	− 0.29	1.11	− 2.51	1.93	0.797
%EWL	− 0.16	0.08	− 0.33	0.01	0.065
Time elapsed since intervention	0.02	0.13	− 0.24	0.28	0.872
Post-BCS complications					
No complications (reference)					
Dehiscence debridement and sutures	− 6.14	3.34	− 12.81	0.53	0.071
Hematoma	0.02	2.98	− 5.92	5.96	0.993
Seroma	3.55	2.12	− 0.68	7.77	0.098
Seroma drainage	9.96	4.65	0.67	19.25	0.036*
Wound infection antibiotics	− 5.23	4.71	− 14.63	4.17	0.271
Wound infection debridement	− 5.51	2.78	− 11.06	0.04	0.052

^*^Statistically significant (*p* < 0.05)

**Table 5:** Results of multiple linear regression analyses for the effect of age, gender, type of weight loss intervention, type of BCS, %EWL, time elapsed since intervention, and post-BCS complications on skin epidermal thickness

Predictors	Estimate	SE	95% CI		*p*
			Lower	Upper	
(Intercept)	71.14	18.29	34.62	107.65	< 0.001*
Age	− 0.12	0.30	− 0.73	0.48	0.686
Gender					
Female (reference)					
Male	0.17	5.16	− 10.12	10.47	0.974
Weight loss intervention					
Non-surgical (reference)					
Surgical	− 0.13	7.77	− 15.64	15.39	0.987
Type of body contour surgery					
Abdominoplasty (reference)					
Breast reduction	− 3.70	3.11	− 9.90	2.50	0.238
%EWL	0.06	0.24	− 0.41	0.53	0.794
Time elapsed since intervention	− 0.66	0.36	− 1.38	0.06	0.072
Post-BCS complications					
No complications (reference)					
Dehiscence debridement and sutures	− 6.46	9.34	− 25.12	12.19	0.491
Hematoma	30.32	8.32	13.72	46.93	0.001*
Seroma	− 4.53	5.91	− 16.33	7.28	0.446
Seroma drainage	30.50	13.01	4.52	56.47	0.022*
Wound infection antibiotics	11.24	13.17	− 15.05	37.53	0.396
Wound infection debridement	8.14	7.77	− 7.37	23.66	0.299

^*^Statistically significant (*p* < 0.05)

## Discussion

To our knowledge, this is the first study that compares the histologic characteristics of skin in prospectively collected biopsies from patients undergoing body-contouring surgery (BCS) following both surgical SMWL and NSMWL. Various definitions of MWL have been reported in the literature, including the criterion used in this study, which defines MWL as a %EWL of ≥ 50% [17]. Other commonly referenced definitions include an excess body mass index loss (%EBMIL) of ≥ 30%, achieving a postoperative BMI of < 30 in patients with a preoperative BMI > 35, and a reduction of 50–100 pounds in body weight [2, 18].

MWL is commonly associated with the depletion of subcutaneous fat and contraction of the adipocutaneous envelope. This process varies among individuals: some patients exhibit better skin tone with successful tissue contraction, while others experience failure in soft tissue retraction, leading to excessive, hanging loose skin. This condition negatively impacts patients’ physical activity and psychological self-acceptance and is also associated with a higher incidence of skin lymphedema and infection [19]. Factors presumed to influence the ability of the adipocutaneous envelope to contract include the rate and amount of weight loss, as well as age, smoking habits, sun exposure, and genetic predisposition [8, 19]. Despite dramatic weight loss after BMS, patients may remain dissatisfied with their body image due to excess skin. A high proportion of patients after BMS, up to 96%, report experiencing excess skin, leading them to seek BCS [3, 4, 8, 19].

Up to 88% of patients undergoing BMS express a desire to undergo BCS, particularly for abdominal excess skin, which is reported by 60% of these individuals [8]. This desire stems from the need to alleviate the discomfort caused by excess skin and its associated psychological, physical, and social consequences. However, most of these patients do not proceed with BCS, often due to obstacles such as health insurance issues or rejection by a plastic surgeon. Additionally, the inclination to undergo BCS tends to decrease over time following BMS [4, 8].

Patients with SMWL face a higher risk of developing complications after BCS compared to those with NSMWL. A significant increased risk, ranging from 60 to 87% in SMWL, was highlighted in a meta-analysis conducted in 2014 [9]. Additionally, a 2021 meta-analysis reported an elevated risk of 37% in SMWL patients who had a body mass index (BMI) of 30 kg/m^2^ or more before undergoing BCS [10]. Furthermore, a notably higher incidence of post-BCS complications has been observed in SMWL patients with associated medical conditions such as dyslipidemia, diabetes mellitus, hypertension, and metabolic syndrome at the time of undergoing plastic surgery [20].

Moreover, SMWL patients are believed to exhibit specific histological changes in their skin, characterized by more pronounced epidermal weakening and a reduced dermal density of collagen and elastic fibers, compared to patients with and without obesity. However, the underlying causes of these changes are not yet fully understood [8].

This study obtained skin biopsies from abdominoplasties and breast reduction procedures, totaling 80 samples. The abdominal and breast regions were specifically chosen for histological examination, as massive weight loss most significantly affects these areas. Literature reports identify the abdomen as the region most commonly exhibiting hanging excess skin following massive weight loss, followed by the breast and chest regions, arms, thighs, and buttocks [8].

In this study, the mean epidermal thickness of the abdomen was 60.3 μm in the NSMWL group and 56.7 μm in the SMWL group. For breast biopsies, the mean epidermal thickness was 56.1 μm in the NSMWL group and 53.1 μm in the SMWL group. A recent systematic review reported a mean pooled epidermal thickness of 66.8 μm (ranging from 31.3 to 102.4 μm) in the breast region of healthy females, 79.2 μm in the abdomen of healthy females, and 100.4 μm in the abdomen of healthy men (ranging from 47.4 to 111.1 μm in females and 222.6 μm in males), with a trend of lower epidermal thickness in aged skin [21]. The epidermal thickness observed in our study was slightly lower than the reported mean in the review. Notably, the SMWL group exhibited marginally lower mean values than the NSMWL group. Though these differences were not statistically significant, they might suggest a higher degree of skin aging in the SMWL group.

In terms of collagen fiber density in this study, the fractions of collagen in the dermis were 49.5% in the abdomen and 49.6% in the breast for the NSMWL group. In contrast, the SMWL group showed 49.2% in the abdomen and 49.3% in the breast, indicating no significant differences between the groups. However, a notable distinction was observed in the density of elastic fibers. In the abdominal skin biopsies, the SMWL group demonstrated a significantly lower fraction of elastic fibers compared to the NSMWL group (5% vs 6.8%, respectively, *p* = 0.029). In contrast, the breast skin biopsies showed fractions of elastic fibers of 6.3% in the SMWL group and 6.7% in the NSMWL group, with no significant differences noted between the two.

Orpheu et al. [3] conducted a comparison of the collagen and elastic fiber content in the abdominal skin of post-BMS patients with massive weight loss to that of patients that never had obesity. Their findings revealed significantly lower fractions of collagen fibers in biopsies from both the upper and lower abdomen of post-BMS patients compared to patients that never had obesity (46.4–47.6% vs 58.6–52.4%, respectively, *p* = 0.001–0.007). Conversely, they observed significantly higher fractions of elastic fibers in biopsies from the upper abdomen of post-BMS patients (9.5% vs 7.7%, respectively, *p* = 0.004). However, the difference in elastic fiber fractions in the lower abdomen biopsies between post-BMS and patients that never had obesity was not significant (7.2% vs 7.6%, respectively, *p* = 0.187).

The collagen fractions observed in both groups of this study closely align with the collagen fractions reported in post-bariatric patients by Orpheu et al. [3]. This similarity suggests a relative lack of collagen fibers in comparison to healthy patients who have not experienced massive weight loss. Such a trend is akin to the aging process of the skin, which is characterized by a depletion of collagen and a reduction in its tensile strength [11].

Another study by Rocha et al. compared the content of collagen and elastic fibers in abdominal skin samples from SMWL patients to those from patients with obesity. They utilized Picrosirius staining with polarized light for studying collagen and Weigert’s resorcin-fuchsin method for elastic fibers. Their findings showed nearly equal total fractions of collagen in both groups (77% in post-bariatric patient’s vs 75% in patients with obesity). However, they observed significant structural changes in the dermis, notably a reduction in thick, well-structured collagen fibers and an increase in loosely arranged, thin collagen fibers in SMWL patients. Additionally, there was an increase in elastic fibers in SMWL patients compared to patients with obesity (1.3% vs 0.8%, respectively) [7].

Our study observed a lower likelihood of seroma occurrence in the surgical group, as indicated by the surgical estimated OR of − 0.13 and the negative association with seroma at − 4.53. However, when seroma was present, it correlated with higher requirements for drainage (30.50) and an increased incidence of hematoma (30.32). These results highlight the complex interplay between surgical outcomes and post-BCS complications. While no significant direct correlation was found between skin epidermal thickness and these complications, the nuanced roles of physiological and anatomical factors, including skin properties, warrant further investigation. This underscores the necessity of a multifaceted approach to understanding and managing postoperative complications in BCS, considering the intricate connections between surgical techniques, patient characteristics, and skin healing and physiology.

The literature provides limited data on skin structural changes associated with MWL. Nevertheless, both the available literature and our findings indicate that MWL generally leads to a depletion of collagen content or a reduction in the structured collagen fibers compared to normal skin. The behavior of elastic fiber content, however, appears to vary; it may either decrease or increase, and these changes can differ across various body regions. Additionally, the diverse methodologies employed for identifying the fractions of collagen and elastic fibers in different studies might contribute to the variability and lack of uniformity in the data.

### Limitations

This study represents the first to compare and analyze skin structural changes following MWL, contrasting outcomes from bariatric surgery with those resulting from non-surgical weight loss methods. Key areas of focus included epidermal thickness, dermal collagen content, and dermal elastic fiber content. These insights may aid in predicting and understanding the skin changes associated with massive weight loss. However, the study is not without limitations. These include the absence of longitudinal follow-up to observe changes in the skin over time, a relatively small sample size—a challenge due to the difficulty in assembling a large cohort of individuals with non-surgical massive weight loss—and the lack of a control group comprising normal skin from patients without obesity. Additionally, a notable limitation is the unknown type of dietary regimen followed by the participants in the NSMWL group, which could influence the skin’s response to weight loss and thus affect the study’s outcomes.

## Conclusion

The SMWL group demonstrated a significant reduction in the content of elastic fibers in the skin of the abdomen compared to the NSMWL group. However, in the breast skin, the differences in elastic fiber content between the two groups were not significant. While the collagen content did not show significant differences in either the breast or abdomen regions between the two groups, it is noteworthy that the collagen content in both groups was reduced compared to the normal skin data available in the literature.

## Data Availability

Data is available with the corresponding author.
